# Date Seeds: A Promising Source of Oil with Functional Properties

**DOI:** 10.3390/foods9060787

**Published:** 2020-06-16

**Authors:** Abdessalem Mrabet, Ana Jiménez-Araujo, Rafael Guillén-Bejarano, Rocío Rodríguez-Arcos, Marianne Sindic

**Affiliations:** 1 Instituto de la Grasa, Consejo Superior de Investigaciones Científicas (CSIC), Campus Universitario Pablo de Olavide, Edificio 46, Ctra. de Utrera, 41013 Seville, Spain; abdessalem_mr@yahoo.fr (A.M.); rguillen@ig.csic.es (R.G.-B.); rrodri@ig.csic.es (R.R.-A.); 2 Department Agro-Bio-Chem, University of Liege—Gembloux Agro-Bio Tech, Passage des Déportés 2, 5030 Gembloux, Belgium; marianne.sindic@uliege.be

**Keywords:** date seed oil, seed oil extraction methods, applications, chemical composition, physicochemical characteristics, phytochemicals

## Abstract

The cultivation of the date palm (*Phoenix dactylifera* L.) is the main activity and source of livelihood for people from arid and semiarid regions of the world. Date production is increasing every year. In addition, pitted date exportation is rising and great amounts of date seeds are produced. This biomass represents a problem for manufacturing companies. At the moment, date seeds are normally discarded or used as animal feed ingredients. However, this co-product can be used for many other applications due to its valuable chemical composition. Oil is one of the most interesting components of the date seed. In fact, date seeds contain 5–13% oil. Date seed oil contains saturated and unsaturated fatty acids with lauric and oleic as the main ones, respectively. Tocopherols, tocotrienols, phytosterols, and phenolic compounds are also present in significant amounts. These phytochemicals confer added value to date seed oil, which could be used for many applications, such as food product formulations, cosmetics, and pharmaceuticals. This review provides up-to-date data on the different extraction techniques and the chemical composition of date seed oils. The applications of date seed oil have also been reviewed.

## 1. Introduction

The date palm has long been one of the most important fruit crops for southern Mediterranean countries, where dates are the main income source and staple food for some local populations. In addition, this crop is not only a source of income from an economic point of view, but also a key for fixing populations and creating or maintaining centers of life. The cultivation of date fruit has been increasing in recent decades, from 1.05 million hectares of cultivated area and 6.44 million tons of production in 2000 to 1.09 Mha and 8.53 MT in 2018. These data also suggest an important increase in the crop yield [1] since with a similar cultivated area, production has been raised by 25%. Egypt, Saudi Arabia, and Iran were the top three producers in 2018, with nearly half the global production. However, Tunisia is the biggest exporter, and is responsible for 17.6% of the worldwide trade (more than $300 million in 2018).

Date fruit production is not only devoted to its consumption as dried fruit (plain, pitted, or stuffed), but also to an increasing manufacturing industrial sector (puddings, bread, jellies, jams, syrups, etc.). Co-products from the date fruit industry (date flesh and seeds) have become an environmental problem for the growing and processing areas. In fact, tons of date seeds (around 10–15% of date fruit fresh weight) are daily discarded [2,3] as unwanted material or used mainly as animal feed for cattle, sheep, and camels [3]. The literature has reported several works devoted to searching for solutions to this problem.

The date seed is mainly composed of dietary fiber, protein, carbohydrates, phenols [4], and minerals (potassium, magnesium, calcium, phosphorus, sodium, and iron). Such substances perform several functions from a biological point of view, such as antioxidant, antibacterial, and antiviral activities [5].

Date seeds are also a good source of oil (5 to 13%), which is rich in phenolic compounds, tocopherols, and phytosterols [3,6,7,8,9]. Date seed oil has been studied by other authors, and its composition in vitamins, minerals, and fatty acids makes it valuable for food formulations [9,10]. The literature data confirm that date seed oil is an interesting source of important nutrients that have a very positive effect on human health.

The most widely used procedures for oil extraction from a solid plant matrix are cold extraction and conventional Soxhlet extraction, which are straightforward, but slow, and produce large volumes of organic solvents that are released into the atmosphere. New technologies have been developed in the last few years and are based on different principles, namely supercritical (SC) fluid extraction, ultrasound-assisted extraction, etc. These methods are associated with some advantages (remarkable reduction in solvent amount, temperature, and time of extraction), compared to solvent extraction methods.

Because of its nutritional and functional composition, date seed oil should be considered a valuable product with commercial importance in the oil industry. This review provides a relevant literature summary of the chemical composition and extraction methods of date seed oil, as well as its application in food and other non-conventional uses. The conclusions could enhance the use of date seeds as valuable new co-products and improve the socioeconomic conditions of growing and manufacturing areas by increasing the business fabric.

## 2. Chemical Composition of Date Seed Oil

### 2.1. Fatty Acid Composition

As shown in Table 1, the fatty acid profiles of several date seed oils are characterized by the presence of five major fatty acids, but in different amounts (oleic acid (C18:1), linoleic acid (C18:2), palmitic acid (C16:0), myristic acid (C14:0), and lauric acid (C12:0)),which together compose more than 90% of the total fatty acid contents [6,11,12,13]. Some fatty acids were detected in lower amounts, including capric (C10:0), palmitoleic (C16:1), linolenic (C18:3), and gadoleic (C20:1) acids (Table 1). Generally, the fatty acid composition of date seed oil can change depending on variety and ripening stage, as well as extraction method [14]. Date seed oil is a source of saturated (lauric, myristic, and palmitic acids), mono-unsaturated (palmitoleic and oleic acids), and polyunsaturated (linoleic and linolenic acids) fatty acids at about 50, 43, and 8%, respectively [10,15]. Al-Shahib and Marshall [14] reported that the highest percentage of unsaturated and saturated fatty acids were oleic and lauric acids, respectively. Biglar et al. and Besbes et al. [7,16] stated that Iranian (Sayer, Khenizi, Majul, Shekar, Zahedi, Goftar, and Khasuee varieties) and Tunisian (Deglet Nour and Allig varieties) dates had more unsaturated than saturated fatty acids. In fact, oleic acid was the main unsaturated fatty acid (41–48%), while the main saturated fatty acid was lauric (17.8%) for the Deglet Nour cultivar and linoleic acid for the Allig cultivar (15%). Furthermore, the degree of unsaturation of these date seed oils is lower than that of common olive oils. Therefore, date seed oil is a good source of oleic acid and the content of this fatty acid is similar to the content found in rice bran oil [17]. According to the literature, when oleic acid is followed by lauric, linoleic, or palmitic acid, the oil is considered as oleic–lauric, oleic–linoleic or oleic–palmitic type, respectively. For example, Al-Hooti et al. [18] stated that United Arab Emirate date seed oils were of the oleic–linoleic or oleic–linolenic types. In another study, Iranian date seed oils were defined as oleic–lauric type [19]. Suresh et al. [20] reported that Omani date seeds oils can be considered as oleic–myristic type.

Generally, oils with high oleic acid contents are of great interest due to their high stability and their nutritional importance. Oleic acid is recognized as one of the most important unsaturated fatty acids in human food because of its preventive effects on distinct heart vascular diseases, its low saturation level, and its potential for reducing cholesterol in the blood, as well as its high oxidative stability [12]. It is widely accepted that dietary oil which is rich in unsaturated fatty acids prevents cardiovascular and inflammatory diseases [21]. Moreover, various studies point out the preventive effect of lauric acid on prostatic hyperplasia development [22], its healthier characteristics compared to trans-fatty acids [23], and its antimicrobial properties, which inhibit the growth of microbes and their production of toxins [22,23,24].

### 2.2. Tocopherol and Tocotrienol Compositions

Tocols (tocopherols and tocotrienols) (Table 2) are present in the unsaponifiable fraction of vegetable oils [31]. Tocopherols and tocotrienols (commonly known as vitamin E) are important for human health due to their lipoperoxyl radical scavenging activities [32,33]. They are known to be very efficient natural antioxidants, which protect biological membrane components. Watson and Preedy [34] showed that the benefits of tocotrienols are significantly greater than α-tocopherol. In addition, tocols also protect oil from free radical damage, thus contributing to their stability. They are more effective than the synthetic antioxidant butylated hydroxytoluene (BHT) [35]. Tocopherols exhibit biological functions such as neuro-protective, anticancer, anti-inflammatory, cardioprotective, immunostimulatory, antidiabetic, hepatoprotective, and nephroprotective activities [36,37].

These natural antioxidants vary from one vegetable oil to another. Different forms of tocols (α-, β-, γ-, and δ-tocopherols and α-, β-, γ-, and δ-tocotrienols) are found in the main sources of vegetable oil (soybean, sunflower, and peanut) [38]. The predominance of one tocol or another depends on the origin of the oil [39]. The average tocol (tocopherols and tocotrienols) content in date seed oil is 74.1 mg/100 g [9]. This value is higher than those of olive oil and *Phoenix canariensis* seed oils, (23.39 and 50 mg/100 g oil, respectively) [9,40], but similar to that of *Chamaerops humilis* seed oil (74 mg/100 g) [41]. Recent research has characterized the tocol family profiles in date seed oil. Fahad et al. [36] reported that tocotrienols are the major tocols in date seed oil from all the cultivars tested (45%). Nehdi et al. [11] showed that date seed oil from six Saudi Arabian cultivars contained seven tocols, namely α-tocopherol, β-tocopherol, γ-tocopherol, δ-tocopherol, α-tocotrienol, γ-tocotrienol, and δ-tocotrienol. However, oils from different varieties or origins had different qualitative and quantitative tocol compositions [10,28,42].

### 2.3. Sterol Composition

The level of phytosterols in vegetable oils is used for the determination of the oil quality and for detecting alterations [44,45]. In general, phytosterols are present in oils in their esterified forms. Sterols, along with tocols, have been shown to be major components of the unsaponifiable fraction.

The literature reported that the major components of date seed oil sterols were β-sitosterol, campesterol, and Δ5-avenasterol [25]. Other minor sterols were cholesterol, stigmasterol, Δ5,24-stigmastadienol, Δ7-avenasterol, and Δ7-stigmastenol [9,25] (Table 2).

According to Laghouiter et al. [28], the sterol contents varied between 4.70 and 8.45 mg/g. These values are similar to other seed oils, such as soybean (9 mg/g) and rapeseed (5 mg/g) [46]. This content is higher than in other species of date seeds, such as *P. canariensis* seed oil (3.36 mg/g oil) and palm kernel oil (1.05 mg/g oil) [25]. Sterol components are important for the functional properties of oils, especially for resistance to oxidation. Consequently, they could have many health benefits. Tapiero et al. [47] have shown that sterols from vegetable oils decreased total and low density lipoprotein (LDL) cholesterol levels in humans by inhibiting cholesterol absorption from the intestine.

### 2.4. Phenolic Compounds

The antioxidant activity may be partly due to some compounds other than tocopherols and sterols, e.g., phenolics [48]. Phenolic compounds are also present in the unsaponifiable matter, or “minor constituents” of oils (Table 2). Boukouada et al. [43] indicated the possible presence of antioxidant phenolic compounds in all date seed oils. On average, the total phenolic content of date seed oil ranged from 0.64 to 1.27 mg/g. However, the phenolic content and antioxidant activity differed according to the cultivar [17]. A study was conducted by Besbes et al. [25] regarding the phenolic compounds present in Tunisian date seed oil (Deglet Nour and Allig varieties). These results showed that the oils extracted from the seeds of Deglet Nour and Allig contained eight phenolic compounds in different amounts (hydroxytyrosol, protocatechuic acid, tyrosol, gallic acid, caffeic acid, p-coumaric, oleuropein, and 3,4-dihyroxyphenylacetic acid). They also concluded that the higher stability of Deglet Nour oil was related to the high amount of phenolics present in Deglet Nour oil [49]. Date seed oil showed a significantly higher concentration of phenolic compounds than olive oil and can be a good source of natural phenolic compounds [49]. Phenolic compounds play an important role in seed oils regarding flavor, shelf-life, and resistance to oxidation. Reduced oil stability could be caused by a higher peroxide content following the extended duration and temperature of extraction, but better oxidative stability could be related to an increase in phenolic compounds.

## 3. Physicochemical Characteristics of Date Seed Oil

The oil content in date seeds varied depending on the date variety, harvest location, particle size, and extraction method. For example, the oil contents in seeds from Tunisian cultivars (Deglet Nour and Allig), were determined as 10.9 and 12.67% [6], the Algerian cultivars Degla-Baïdha and Tafezouine contained 5.6 and 5.4% of oil [43], cultivars from Sudan (Albarakavi, Alqundeila) had 10.5 and 7.8% [50], and in three Iranian date varieties (Kabkab, Shekar, Shahabi), 8.2, 8.3, and 8.4% were detected, respectively [29]. These values revealed that date seed oil contents are important compared to other seed oils, such as corn (3.0–6.5%) [51]. In addition, this oil could be considered as a source of compounds (fatty acids, phenolic derivatives, phytosterols, and tocopherols) with pharmacological and food interest.

Concerning its physicochemical properties, date seed oil is liquid at room temperature, yellowish in color, and has a pleasant odor. Its content of carotenoids, 2–5 mg/Kg [9,41], makes it suitable for margarine production [52].

There are several parameters that define the quality of plant oils. Iodine value (IV) is a valuable characteristic used to assess the unsaturation of oils and their stability in industrial applications, but it does not define a specific fatty acid composition [53,54]. High IV in oils is indicative of the presence of many unsaturated bonds and of containing more unsaturated fatty acids [55]. Additionally, oil IV is known to predict and reflect the oil’s drying property. Drying oils have an IV of about 190, semi-drying near 130, and non-drying around 100. Therefore, date seed oil, with an IV lower than 100, is classified as non-drying [56]. Dehdivan and Panahi [19] reported that the IV of Iranian date seed oil was in the range of 46–65 g/100 g oil. Similarly, Besbes et al. [6] described the IV for Tunisian date seed oil as 44–61 g/100 g oil, and Nehdi et al. [41] determined a value for *C. humilis* (55.58 g/100 g oil), which was weaker than those of other palm oils, such as *P. canariensis* (77.66 g/100 g oil) [9].

Acidity is another quality parameter [55] and it is defined as the number of milligrams of potassium hydroxide necessary to neutralize the free acids in 1 g of sample. The acidity value, which is an index of free fatty acid content and also an indicator of the edibility of oil and its suitability for industrial use, was found to be lower in *P. dactylifera* L. (1.83 mg/g) than in watermelon seed oil (6.10 mg/g) [57]. This value for *P. dactylifera* L. indicates negligible amounts of free fatty acids and is closely comparable to melon seed oil [58]. Bouhlali et al. [30] pointed out that the acidity value of Moroccan date seed oil was between 1.083–1.813 mg KOH/g, similar to that of the olive oil analyzed by Borchani et al. (2010) [59], which means that date seed oil could be considered as an edible oil.

The saponification value (SV) is a measurement of the fatty acid molecular weight. It gives information about the nature of fatty acids and depends on the average molecular weight of these fatty acids [60]: SVs greater than or equal to 180 mg KOH/g have low molecular weight fatty acid and vice versa [61]. In addition, it is used to predict the type of triacylglycerols in the oil. According to Nehdi et al. [62], the high SV of date seed oil indicates a very high content of low molecular weight triacylglycerols. This is desirable characteristic for the use of date seed oil as biodiesel and in the production of liquid soaps and shampoos [12,63,64,65]. In general, the average SV of date seed oil ranges from 198 to 228 mg KOH/g oil [27,56,66]. These values were comparable to the values of some edible oils, such as palm oil (196–205 mg KOH/g), corn oil (187–196 mg KOH/g), and palm kernel oil (247 mg KOH/g) [51].

Although its extraction yield is lower than that of other oilseeds, the physicochemical characteristics of date seed oil reveal that date seeds could be valued as an oil source. This oil could have culinary or industrial applications but considering its phytochemical composition, it could also be of great interest as a functional oil.

## 4. Date Seed Oil Extraction

### 4.1. Conventional Extraction: Soxhlet Extraction

Organic solvent extraction is the most common method used for extracting oil. Many researchers have reported the use of conventional extraction for date seed oil with different solvents (petroleum ether, hexane, chloroform, methanol, etc.) using the maceration and Soxhlet methods. These solvents have characteristics such as easy removal by evaporation from the extracts, high solvent–solute ratio, oil viscosity, and polarity [67,68]. The choice of the solvent type depends on its capacity to drive the extraction process and ensure maximum yield. In the organic solvent method, the date seed is cleaned, dried, and ground to rupture the oily cells, making the oil available to the solvent. The extraction yield is related to the degree of accessibility of the solvents to the oil-containing cells [69]. In general, Soxhlet extraction is the most common technique for oil seed extraction (Table 3) and is generally used as a reference.

During solvent extraction, diffusion is the main mechanism. The seed and solvent come into contact; the solvent diffuses through the seed mass and extracts the available oil from broken oily cells. The oil is then separated from the solvents using a rotavapor [76]. Many researchers and industrialists have used hexane as the extraction solvent due to its availability, great oil extractability (98%), and easy operation.

Although a higher oil extraction yield is obtained by Soxhlet extraction, this technique gives much more complex extracts and has some drawbacks which are especially detrimental to the pharmaceutical and oil industries, such as long operation time, a high consumption of solvent, a large volume of organic solvents which are released into the atmosphere, and the presence of solvent residue in the final product.

### 4.2. Innovative Extraction Methods

The use of organic solvents for the extraction of bioactive compounds is subjected to increasingly strict regulations, which are aimed at ensuring the protection of human health and the environment [77]. Nowadays, the need for new and clean technologies is peremptory. These technologies have several advantages, such as reducing costs and duration (completed in minutes), and the improved quality of the extracted oil.

#### 4.2.1. Supercritical Fluid Extraction (SFE)

SFE has been studied as a clean alternative to solvent extraction [78] due to its many positive characteristics compared to traditional extraction. Consequently, supercritical fluid extraction is now widely used.

Recently, the pharmaceutical and food industries have been using SFE for obtaining valuable compounds from solid matrices. SFE has been classified as a green and ecological method, mainly due to its use of supercritical carbon dioxide (SC-CO_2_) as a solvent. It is biologically safe with no solvent residue in the final product, especially in very sensitive fields like the pharmaceutical, cosmetic, and food industries. Therefore, SC-CO_2_ could be considered a promising technique for the extraction of vegetable matrix oils without inducing degradation [79,80]. Thanks to the high diffusion capacity and high solvating power of CO_2_, SC-CO_2_ is considered the most efficient method for the extraction of compounds from plant matrices and the most commonly used solvent in SFE [81,82]. In addition, differences in CO_2_ solubility can be adjusted by controlling factors such as pressure and temperature. Aris et al. [73] identified pressure as the dominant factor for increasing the solubility of a solute, which results in a higher extraction yield of date seed oil. Indeed, increasing the pressure enhances the density of CO_2_, which increases the extraction yield [83]. However, Takadas and Doker [68] showed that when the temperature is increased, the vapor pressure is increased as well, leading to a decrease in the density of CO_2_, which could decrease the oil yield. In another study done by Mehdi et al. [75] on Algerian date seed oil extracted using the SC-CO_2_ method, the effects of the different parameters (pressure, temperature, and particle size) on the extraction yield, as well as the fatty acid profile, were investigated. The results revealed that the pressure and the interaction between the pressure and temperature had a significant positive effect on the extraction yield of date seed oil. The fatty acid profile (49.85% saturated fatty acids, 42.75% monounsaturated, and 7.55% polyunsaturated) was similar to Soxhlet extraction. Therefore, SC-CO_2_ extraction is considered a very promising technique for obtaining date seed oil and giving an added value to this by-product. It constitutes a safe means for obtaining edible oils without using any contaminating organic solvent with similar yields to Soxhlet extraction. The limitation of SC extraction comes mainly from expensive equipment, which increases the cost compared to conventional extraction.

#### 4.2.2. Ultrasonic-Assisted Extraction (UAE)

Another new extraction technology that may improve the extraction of oil from seeds is known as sonication-assisted or ultrasound-assisted solvent extraction (UAE). Its emergence as a novel green technology has attracted attention due to its role in environmental sustainability. UAE is an innovative technique that makes use of ultrasonic sound waves of high intensity and high frequency to increase vibration and alter the physical and chemical properties of plant tissues (the disruption of plant cell walls), thereby enhancing the contact between solvent and plant material and facilitating the release of extractable compounds [68,84]. When ultrasound is transmitted in a liquid, the phenomenon of cavitation is induced, which leads to cell disruption. The propagation and interaction of sound waves alter the physicochemical properties of materials, causing a series of compressions and rarefactions in the solvent and inducing the creation of micro bubbles in the liquid. This phenomenon is known as “acoustic cavitation” and provides cell wall destruction. In this way, UAE increases the permeability of the solvent into the plant tissues by physical effects: cavitations and the formation of cracks and microfractures on seed surfaces [72].

The utilization of UAE represents an innovative way of thus enhancing the extraction efficiency of bioactive substances from plants and seeds and increasing oil yield. Babaei et al. [85] suggested that UAE could be recognized for reducing extraction time and also for improving production efficiency. The extraction efficiency of UAE of seed oil was equal to or better than that of conventional extraction (Soxhlet) but with a remarkable reduction in extraction time [86]. This fact has also been tested for extracting oil from date seeds [72], and it was concluded that UAE reduces the extraction time (75% less compared to the Soxhlet method), making it a more efficient method due to its low energy consumption (76.64% less than Soxhlet).

Working with soybean germ, Cravotto et al. [87] concluded that an 8.6% oil yield can be obtained by Soxhlet, instead of 17.7% obtained by UAE. In this case, an acceleration of extraction kinetics and an increase in yield were observed. The same conclusion was reached when working with papaya seeds [88], where only 30 min were needed to reach a 76.1% oil yield, compared to 12 h to produce a 79.1% yield by Soxhlet. Similar results were found by studying the extraction of tobacco seed oil [89]. Recently, Khoei and Chekin [90] compared the conventional and UAE methods for rice bran oil extraction. From their study, they found that aqueous UAE gave a similar extraction yield (20−22%) to the Soxhlet method using hexane as an extracting solvent.

However, UAE presents a disadvantage. When applied to oil extraction, the high energy level in the medium leads to an increase in free radical generation, thus decreasing the oxidation stability of the extracted oil [91]. This effect was observed during the extraction of grape seed oil [92], where a significantly higher amount of free radicals was quantified after 7 days of storage in UAE oil than in Soxhlet oil. Free radicals trigger oxidation chain reactions, which are responsible for the characteristic flavor of oxidized oils. Working with sunflower oil, Chemat et al. [93] identified limonene, aldehydes, and 2-methylfuran in the UAE oils. These compounds, characteristic of a rancid flavor, negatively affect the shelf-life and consumer acceptance of oils [92]. This sonication side effect seems to be directly related to the seed/solvent ratio during extraction. Oxidative stability suffers drastic reductions when this ratio is higher than 10 [92], which contributes to supporting the strategy of using the smallest amount of solvent possible.

## 5. Potential Applications of Date Seed Oil

### 5.1. Culinary Uses

Date seed oil presents a valuable chemical composition and physicochemical characteristics for its use as edible oil. It is rich in oleic acid, which confers nutritional importance. Its content of saturated fatty acids and the presence of many other antioxidants (phenolic compounds, tocols, carotenoids, etc.) make it highly stable against oxidative rancidity [9] and thermal treatments [7], making date seed oil suitable as a cooking, frying, or seasoning oil, or even as an alternative to palm olein [11]. Its content of carotenoids is adequate for margarine production since they provide a natural yellowish butter-like color without the addition of synthetic colorants [9]. Basuny and Al-Marzooq [26] replaced conventional corn oil with date seed oil for producing mayonnaise, which had higher sensory characteristics than the control. However, any applications for human consumption must be investigated for their complete safety.

### 5.2. Health Beneficial Effects

Date seed oil has the potential to be used in cosmetic and pharmaceutical practices as well. Date seed oil can protect against UV-B and UV-A radiation due to its absorbance spectrum of UV radiation, which is responsible for most cellular damage to skin [49], so date seed oil may be used in the formulation of UV protectors [9]. Ines et al. [94] studied it as a chemopreventive agent using a normal human epidermal keratinocyte model, concluding that this oil was able to prevent oxidative damage caused by H_2_O_2_ exposure. Besides, it did not show any toxic effect on cells at a dose as high as 30 μg/mL. These same authors [95] irradiated human skin samples with UV-B and observed that skin cultures with date seed oil had four times lower DNA damage than those without it for the same irradiation level. The authors related these effects to the content of phenolics and tocols present in this photoprotective oil. In fact, Lecheb and Benamara [96] formulated a cosmetic cream containing date seed oil and an aqueous seed extract. The optimized cream had similar spreadability, viscosity, and rheological behavior to other commercial creams, with the advantage of the substitution of synthetic components with natural ones. These bio-creams could have a great acceptance by consumers, who are more and more concerned about the use of chemicals in cosmetics [17].

The defense against reactive oxygen species (ROS), which are responsible for cellular oxidative damage, is not limited to skin care. In another study, Ben Abdallah et al. [97] tested the effects of date seed oil on human sperm motility and viability after in vitro H_2_O_2_-induced oxidative damage. They found that date seed oil has a protective effect on both sperm parameters, especially after 24 h incubation. All these studies highlighted the importance of incorporating natural products with high contents of antioxidants into the normal human diet and also into cosmetic and/or dietary supplements. Date seed oil can be considered a promising dietary product with proven antioxidant effects, particularly if extracted by non-contaminating and environmentally friendly procedures.

The chemical composition of date seed oil obtained by slow pyrolysis has been studied by GC/MS [98]. Compounds such as triterpenoids and several steroids, identified in this oil, could be of great interest due to their adaptogenic and anabolic activities. The content of stearic, palmitic, and oleic acids in this oil make it suitable for the formulation of anti-inflammatory pharmaceutical preparations, not as active ingredients, but as coadjuvants, since they enhance the percutaneous absorption of non-steroidal anti-inflammatory drugs [99].

### 5.3. Feedstock for Industrial Processes

Date seed oil shows outstanding promise, not only in the production of some functional food products and pharmaceuticals, but also as a renewable resource. Azeem et al. [100] concluded that this oil is also adequate for the production of biodiesel because of its low content of free fatty acids. A study conducted by Al-Zuhair et al. [101] using different catalysts (NaOH and Novozym^®^435), concluded that the yields were similar for both catalysts. However, the selectivity of NaOH was high toward transesterifying trans-9-elaidic acids compared to other acids, whereas Novozym^®^435 equally transesterified most acids present in the oil sample. Another industrial application that has been explored is as feedstock for the synthesis of poly(3-hydroxybutyrate) (PHB), a biodegradable polyester that could replace contaminating plastics [102]. The microorganism *Cupriavidus necator* can use date seed oil instead of edible vegetable oils as a sole carbon source and produce PHB at a reasonably good concentration and accumulation. The PHB obtained from date seed oil demonstrated physicochemical characteristics similar to standard PHB. The use of oils which are not expensive and do not compete with food as feedstock for the production of biodiesel and other chemicals is highly recommended. This is the case of the date pit, an abundant agro-food waste product with the appropriate chemical composition for these purposes.

## 6. Conclusions

Date seed oil is remarkable for its content of monounsaturated fatty acids, especially oleic acid. It is an excellent source of important lipid-soluble antioxidant compounds, such as phenols, tocopherols, and phytosterols, which play an important role in reducing the risk of many diseases. With the increasing availability of date seeds, which constitutes a troublesome waste product, their oil extraction may be economically beneficial and should be considered an opportunity for future venture. The extraction could be achieved by various methods, including conventional (Soxhlet) and non-conventional ones (UAE and SC-CO_2_), whose efficiency and oil quality vary depending on several parameters. The old traditional methods were largely inefficient and contaminating, which results in the need for launching new, more environmentally friendly methodology, such as UAE and SC-CO_2_. Based on its chemical and functional composition, date seed oil can be presented as a novel oil with interesting and varied applications, from human food (cooking, frying, seasoning, or shortening oil) to pharmaceutical and cosmetic applications, and even biodiesel or bio-plastic production. Date seeds would evolve from waste to feedstock for several industrial activities, which would support the economic and social development of date fruit producing and manufacturing areas.

## Figures and Tables

**Table 1:** Fatty acid profiles of date seed oil expressed as percentage of total fatty acid quantified.

References	Besbes et al. (2004) [25]	Basciny et al. (2011) [26]	Dehdivan et al. (2017) [19]	Nehdi et al. (2018) [11]	Raza et al. (2019) [27]	Laghouiter et al. (2018) [28]	Akbni et al. (2012) [29]	Bouhlali et al. (2017) [30]
Saturated								
**Capric acid (10:0)**	0.80	0.25	0.2–0.5	0.71	0.48–0.55	nd ^1^	0.47–0.50	nd
**Lauric acid (12:0)**	17.8	35.31	14–15.8	10.36	25.7–30.8	6.6–25.4	25.6–30.8	16.7–20.3
**Myristic acid (14:0)**	9.84	0.04	10.6–10.9	10.44	6.9–16.7	9.3–19.3	13.3–16.9	10.2–12.3
**Palmitic acid (16:0)**	10.9	12.58	10.8–11.8	12.83	11.9–13.1	9.6–17.6	11.9–13.1	9.8–10–9
**Stearic acid (18:0)**	5.67	3.3	3–3.4	5.56	1.8–2.3	0.8–3.4	1.8–2.3	2.9–3.7
Monounsaturated								
**Palmitoleic acid (16:1)**	0.11	nd	0.2–0.4	nd	nd	0.09–1.66	nd	0.05–0.09
**Oleic acid (18:1)**	41.3	39.5	48.1–50.5	51.45	31.5–37.6	37.8–52.5	31.5–37.6	44.9–48.4
**Gadoleic acid (20:1)**	nd	nd	0.2–0.4	0.37–0.52	nd	0.16–0.65	nd	0.26–0.40
Polyunsaturated								
**Linoleic acid (18.2)**	12.2	8.2	7.7–8.2	7.2	4.4–6.9	5.7–10.4	4.4–7.0	8.30–9.02
**Linolenic acid (18:3)**	1.68	0.81	0.4–0.7	nd	nd	0.10–0.59	nd	0.09–0.21

^1^ nd: not detected.

**Table 2:** Unsaponifiable components of date seed oil.

	Content	Composition	References
Tocols (mg/100 g)	32–74	α-tocopherol (74%), (β+γ)-tocopherol (40.56%), δ-tocopherol (28.41%)	Laghouiter et al. (2018) [28]
	54.65	α-tocotrienol (63.28%), γ-tocopherol (17.72%), γ-tocotrienol (11.84%), δ-tocotrienol (3.60%), α-tocopherol (1.85%), β-tocopherol (1.7%)	Fahad et al. (2017) [42]
	0.053–0.143	α-tocopherol (51.02%), (β+γ)-tocopherol (30.61%), δ-tocopherol (12.24%)	Boukaouada et al. (2014) [43]
	1.01–1.86	α-tocopherol (52.54%), α-tocopheryl acetate (27.68%), γ-tocopherol (19.76%)	Habib et al. (2013) [10]
	24.97–42.08	α-tocopherol (38.8%), γ-tocopherol (5.4%), δ-tocopherol (2.4%)	Besbes et al. (2004) [25]
	51.54	α-tocotrienol (66%), γ-tocopherol (10.3%), γ-tocotrienol (4.6%), δ-tocopherol (1%), β-tocopherol (0.9%), α-tocopherol (0.6%)	Nehdi et al. (2010) [9]
	70.75	α-tocotrienol (30.19%), γ-tocopherol (23.61%), γ-tocotrienol (19.07%), α-tocopherol (17.52%), δ-tocotrienol (5.89%), β-tocopherol (2.42%), δ-tocopherol (0.9%)	Nehdi et al. (2018) [11]
Sterols (mg/100 g)	300–350	β-sitosterol (80%), campesterol (10%), Δ5-avenasterol (4.5%), stigmasterol (2.42%), cholesterol (0.96%), Δ5,24-stigmastadienol (0.41%)	Besbes et al. (2004) [25]
	470–845	ND ^1^	Laghouiter et al. (2018) [28]
	336	β-sitosterol (76%), campesterol (8.89%), Δ5-avenesterol (8.79%), Δ5,24-stigmastadienol (2.73%), Δ7-avenasterol (1.18%), stigmasterol (1.09%), Δ7-stigmastenol (0.79%), cholesterol (0.42%)	Nehdi et al. (2010) [9]
Phenols (mg/kg)	220.3–520.8	Hydroxytyrosol (10.21%), protocatechuic acid (9.62%), tyrosol (8.10%), caffeic acid (4.95%), gallic acid (4.11%), p-coumaric acid (0.26%), oleuropein (0.18%)	Besbes et al. (2004) [25]
	640–1270	ND	Boukaouada et al. (2014) [43]

^1^ ND: not detected.

**Table 3:** Date seed oil recovery based on extraction methods.

Method	Conditions of Extraction	Conclusions	References
Soxhlet	Extraction yield 5% (chloroform, hexane), 4 h Extraction yield 1% (propanol, methanol), 4 h	The yield is better with non-polar solvents (n-hexane, chloroform) compared to polar solvents (methanol and propanol)	Ali et al. (2015) [70]
Soxhlet	Extraction yield 4.44%, hexane, 8 h	The ultrasound- and microwave-assisted extractions reduced the extraction time compared to Soxhlet and of extraction yield compared to maceration	Ben-Youssef et al. (2017) [71]
Ultrasound	Extraction yield 6.18%, hexane, 15 g, 20 °C, 30 min		
Microwave	Extraction yield 4.74%, methyltetrahydrofuran (MeTHF), 15 g, 30 min		
Maceration	Extraction yield 4.04%, MeTHF, 15 g, RT, 30 min		
Soxhlet	Extraction yield 8.5%, hexane, 50 g, 78 °C, 3 h	The extraction by ultrasound shortened extraction time when compared to Soxhlet, reduced energy consumption, and had higher yield.	Jadhav et al. (2016) [72]
Maceration	Extraction yield 4.2%, hexane, 50 g, RT, 3 days		
Ultrasound	Extraction yield 8.5% hexane, 50 g, 20 °C, 45 min		
SC-CO_2_	Extraction yield 3%, 5 g, 40 min, 70 °C	In this case, the oil yield was very low, probably due to several compatibilities of CO_2_ with the oil.	Aris et al. (2013) [73]
Soxhlet	Oil yield 8.5%, 120 min, hexane	The oil composition indicated the presence of low molecular weight saturated fatty acids.	Ali et al. (2015) [70]
Soxhlet	Oil yield 9.78, 8, and 9.5%, methanol, ethanol and acetone, respectively	The best conditions were found using methanol 15 °C above its boiling point, particle size range of 0.212–1 mm, 4 h.	Al-Sumri et al. (2016) [74]
SC-CO_2_	Oil yield 14%, 250 bar, 333 K	The pressure and the interaction between the pressure and temperature had a positive significant effect on the extraction yield	Mehdi et al. (2019) [75]
